# Preclinical evaluation of the epithelial sodium channel inhibitor AZD5634 and implications on human translation

**DOI:** 10.1152/ajplung.00454.2021

**Published:** 2022-09-13

**Authors:** Annika Åstrand, Emily Falk Libby, Ren-Jay Shei, Jacelyn E. Peabody Lever, Niroop Kaza, Adegboyega Timothy Adewale, Evan Boitet, Lloyd Edwards, Martin Hemmerling, James Root, Botilda Lindberg, Cecilia Wingren, Anna Malmgren, Juan Sabater, Steven M. Rowe

**Affiliations:** ^1^Research and Early Development, Respiratory & Immunology, BioPharmaceuticals R&D, AstraZeneca, Gothenburg, Sweden; ^2^Cystic Fibrosis Research Center, University of Alabama at Birmingham, Birmingham, Alabama; ^3^Department of Medicine, University of Alabama at Birmingham, Birmingham, Alabama; ^4^Department of Biostatistics, University of Alabama at Birmingham, Birmingham, Alabama; ^5^Department of Cellular, Developmental, and Integrative Biology, University of Alabama at Birmingham, Birmingham, Alabama; ^6^Department of Pediatrics, University of Alabama at Birmingham, Birmingham, Alabama; ^7^Mount Sinai Medical Center, Miami, Florida

**Keywords:** airway surface liquid, ion channels, lung disease, mucociliary clearance

## Abstract

Airway dehydration causes mucus stasis and bacterial overgrowth in cystic fibrosis (CF), resulting in recurrent respiratory infections and exacerbations. Strategies to rehydrate airway mucus including inhibition of the epithelial sodium channel (ENaC) have the potential to improve mucosal defense by enhancing mucociliary clearance (MCC) and reducing the risk of progressive decline in lung function. In the current work, we evaluated the effects of AZD5634, an ENaC inhibitor that shows extended lung retention and safety profile as compared with previously evaluated candidate drugs, in healthy and CF preclinical model systems. We found that AZD5634 elicited a potent inhibition of amiloride-sensitive current in non-CF airway cells and airway cells derived from F508del-homozygous individuals with CF that effectively increased airway surface liquid volume and improved mucociliary transport (MCT) rate. AZD5634 also demonstrated efficacious inhibition of ENaC in sheep bronchial epithelial cells, translating to dose-dependent improvement of mucus clearance in healthy sheep in vivo. Conversely, nebulization of AZD5634 did not notably improve airway hydration or MCT in CF rats that exhibit an MCC defect, consistent with findings from a first single-dose evaluation of AZD5634 on MCC in people with CF. Overall, these findings suggest that CF animal models demonstrating impaired mucus clearance translatable to the human situation may help to successfully predict and promote the successful translation of ENaC-directed therapies to the clinic.

## Graphical abstract

**Figure:**
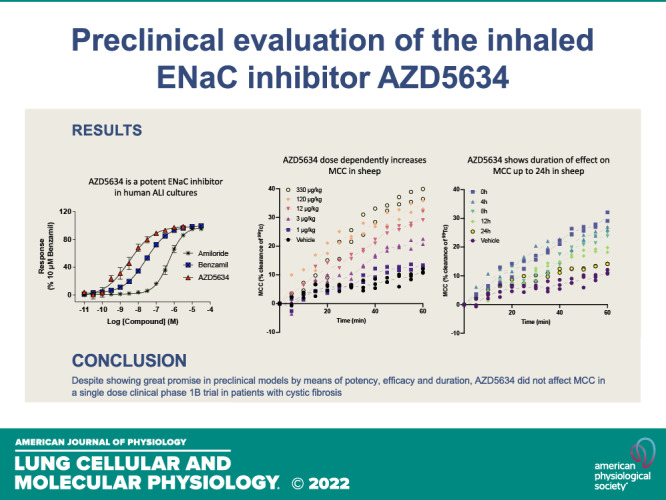

## INTRODUCTION

Cystic fibrosis (CF) is a severe life-limiting disease that results from mutations in the cystic fibrosis transmembrane conductance regulator (CFTR) anion channel (1). Dysfunction in CFTR causes prominent pulmonary disease, including a depleted airway surface liquid (ASL) volume and highly viscous mucus that lead to malfunction of the mucociliary clearance (MCC) apparatus (2, 3). Together with airway acidification and defective bacterial killing (4), among additional defects in host defense seen in people with CF, impaired MCC favors a vicious cycle of chronic infection and inflammation that precipitates progressive and irreversible lung damage and destruction (1).

Strategies to rehydrate the airway mucus have been a primary focus of therapeutic development in CF and encompass approaches such as inhalation of aerosolized hypertonic saline, modulation of CFTR to facilitate fluid secretion in the airway lumen, and targeting of other ion channels involved in ASL regulation. Prominently, inhibition of the epithelial sodium channel (ENaC), which promotes sodium and fluid absorption into the epithelial cell, is being explored as an approach (5–10). ENaC is thought to be aberrantly active in CF (11, 12), with evidence suggesting that this is attributable in part to CFTR dysfunction (13, 14), although the presence of hyperactive ENaC and the role of CFTR remain a matter of debate (15–17). Although therapies that increase CFTR activity have been successfully developed and approved for the majority of people with CF (18), to date pharmacological ENaC inhibitors as a treatment for CF have failed to translate to clinical use despite a number of efforts to advance this approach (9, 10). An advantage of ENaC inhibition is that it has the potential to circumvent CFTR function and *CFTR* mutation class (19, 20) and could thus be applicable to all patients with CF, notably those possessing rare mutations who do not currently have access to approved CFTR modulator therapy (21).

Several ENaC inhibitors have initiated development in CF (9, 22–25). Many of these have relied on preclinical development assays that included inhibition of amiloride-sensitive short-circuit current and augmentation of ASL depth of cultured human epithelial cells derived from CF donors (26–29), followed by estimations of MCC in sheep with or without invoked MCC deficits (26, 27, 29, 30). This has raised some uncertainty as to the predictive potential of these tools in patients with CF, since the full complexities of the CF mucus clearance defect are not present in these model systems, although it is also plausible that the efficacy, potency, and durability of prior ENaC inhibitors were insufficient to impart clinical benefit. As more sophisticated CF animal models have become available that spontaneously exhibit defects in MCC (3, 31, 32), this points to the need to assess the impact of ENaC inhibition on downstream defects of CF pathophysiology in these systems, including mucus clearance (3, 33, 34).

AZD5634 is a novel small-molecule ENaC inhibitor that has a high affinity for ENaC and is not readily absorbed across the airway epithelium. Unlike previous small-molecule ENaC inhibitors, including the pyrazinoyl guanidine compound amiloride and its derivatives, AZD5634 is retained at the apical side of the airway epithelium after inhalation and/or nebulization and does not cause hyperkalemia as its renal clearance is minimal (unpublished information). In addition to blocking ENaC function, AZD5634 is suggested to also inhibit sodium/hydrogen exchangers, facilitating mucus detachment in murine intestine and CF pigs (35). As a result, AZD5634 may be a promising new approach to ENaC inhibition for the treatment of CF. Here, we describe the effects of AZD5634 in normal and CF preclinical model systems in vitro, in vivo, and in situ.

## METHODS

### Evaluation of AZD5634 Potency and Efficacy in Normal Healthy Preclinical Systems

#### Ussing chamber potencies in human and sheep air-liquid interface cultures.

Normal human bronchial epithelial cell (HBEC) cultures from healthy donors were procured from MatTek Corporation (Ashland, MA), grown at an ALI on Transwell permeable supports (0.4 µm polycarbonate membrane, 12 mm insert, and Costar Corning), and used to test the in vitro potency of AZD5634. Using the same protocol, sheep ALI cultures were prepared from primary bronchial epithelial cells (native sheep tracheas were obtained from Öströö Sheep Farm, Sweden, and internally processed at AstraZeneca R&D, Gothenburg, Sweden) to validate AZD5634 potency at sheep ENaC. Experiments were performed using modified Ussing chambers containing carboxygenated Krebs solution (95% O_2_-5% CO_2_) at 37°C. Cell layers were allowed to equilibrate before being voltage-clamped at 0 mV, and then briefly clamped at 10 mV to assess transepithelial electrical resistance. While measuring short-circuit current (*I*_sc_), inhibitors were applied cumulatively before benzamil (10 µM) was added on the final response plateau to which calculated responses were standardized (100%). AZD5634 was tested compared with amiloride and benzamil in a concentration range from 10 pM to 30 µM.

#### Gravimetric measurements of ASL height.

The efficacy and duration of AZD5634, benzamil, and amiloride were observed by means of gravity in HBECs grown under ALI conditions (Donor 231849, Cat. No. CC-2540, Lonza) in a 24-well plate format using the Transwell clear filters (0.4 µm polycarbonate membrane, 6.5 mm insert, and Costar Corning). After 3–4 wk, the HBEC ALI cultures were washed with PBS twice before the experiment was initiated. Thirty microliters of either vehicle or test compound (10 μM) was added apically and inserts were weighed after removal of basolateral fluid as a crude but simple way to measure retention of fluid on the apical side of the airway cultures. The weighing procedure was repeated at 4, 8, and 24 h.

#### AZD5634 rat potency evaluated from renal sodium excretion.

The potency of AZD5634 was also assessed in vivo in rats. Female Wistar rats were anesthetized by means of spontaneously breathing isoflurane (induction concentration of 5% followed by 2%). A catheter was positioned in the carotid artery for blood sampling and mean arterial blood pressure and heart rate were measured. Another catheter was placed in the vena jugularis for continuous infusion of test compound according to a three-step dose design where each step had a duration of 30 min (bolus given over 6 min + infusion over 24 min giving a total of: 0.02−0.2–2.0 μg/kg/min × 30) or vehicle (68 μL/kg/min during bolus and 17 μL/kg/min during infusion, giving a total of 1.6 mL/kg/h) plus a constant infusion of 0.9% NaCl (12 mL/kg/h) to secure urine production. The urinary bladder was catheterized for urine collection. The rats were left to stabilize for at least 30 min after surgery during constant basal infusions before any test compound or vehicle infusion was initiated. Urine was collected over 20-min periods; two at baseline (i.e., preexposure), followed by three 30 min exposures of increasing doses of test compound or vehicle, where urine was collected over the last 20 min for each dose. Additional urine was collected during the washout period (0–20, 20–40, and 40–60 min after cessation of the infusion protocol). Blood samples were collected 5 min before the end of each urine collection period for electrolyte evaluation (iSTAT analyzer, Abbott Laboratories Inc) and plasma concentration of drug (LC-MSMS). Urine production, electrolytes (ABL700, Radiometer Medical ApS, Brønshøj, Denmark, for urine), as well as concentration of test compound (LC-MSMS) were evaluated. Effective concentration at 50% level (EC_50_) was determined from the measured plasma and urine concentrations (free, corrected for protein binding) by using a concentration gradient of sixfold from the distal tubuli (where ENaC is expressed) (36).

#### MCC effects of AZD5634 in healthy sheep.

Twenty-one adult female Florida native sheep (37–54 kg, Fair Meadow Sheep) were used in the present study, once or twice with a washout of at least 1 wk between experiments. Animals were restrained in an upright position in a specialized body harness in modified carts. The head of the animal was immobilized, and after local anesthesia of the nasal passage was induced with 2% lidocaine, the animals were nasally intubated with a standard endotracheal tube (7.5 mm diameter, Mallinckrodt, St. Louis, MO). A flexible bronchoscope was used to guide the tube and verify its position in the trachea. After intubation, the animals were allowed to rest for ∼20 min before administration of any agent.

Nebulized drug or placebo was given as an aerosol where 3 mL volume (compound in 10% EtOH:90% saline) was delivered at a tidal volume of 300 mL. To maximize central deposition in the airways, a dosimeter was used to deliver the compound. The nebulizer was connected to the dosimeter system consisting of a solenoid valve and a source of compressed air (20 psi). The output of the nebulizer was connected to a T-piece, with one end attached to a respirator (Harvard Apparatus Inc., Holliston, MA). The system was activated for 1 s at the onset of the inspiratory cycle of the respirator, which was set at an inspiratory/expiratory ratio of 1:1 and a rate of 20 breaths/min. To evaluate the effect of repeated dosing, AZD5634 was given twice daily for 4 days and MCC was measured 8 h after the 7th dose.

MCC rate was measured by means of aerosolized technetium-labeled sulfur colloid (^99m^TC-SC) and nebulized via the same dosimeter system as for the drug administration. To compare the effects between conditions and vehicle, we also looked at the area under the curve (AUC) generated by the MCC as a percentage over the first 60 min (data not shown). Aerosol ^99m^TC-SC was generated using an Air Life nebulizer (CareFusion, Yorba Linda, CA). Approximately 20 mCi of ^99m^TC-SC in a total of 2 mL of sterile saline was placed in the nebulizer. A tidal volume of 500 mL was used to deliver the ^99m^TC-SC over 3 min. A gamma camera (Dyna Cam, Picker Corp., Northford, CT) integrated with a computer was used to record and analyze the clearance of ^99m^TC-SC every 5 min for 1 or 2 h (only the first hour data are used for analysis of the results).

To evaluate pharmacological half-life of effect, blood (4 mL) was sampled pre-dosing (baseline) and immediately after cessation of dosing plus at 15 min, 30 min, 1, 2, 4, 6, and 24 h post dosing (in total 9 samples per experiment) in EDTA-precoated venojects (BD K2, Becton Dickinson). Blood was kept on ice until centrifuged (4,000 *g*, 5 min, 4°C) and plasma (approximately 2 mL) was transferred to two Eppendorf tubes per sample and frozen at −20°C until analyzed. Sodium and potassium concentrations were analyzed at Mount Sinai Medical Center immediately after sampling and before centrifugation (IRMA TruPoint Analysis System). The plasma samples for analysis of drug concentration were shipped on dry ice to AstraZeneca R&D, Mölndal, Gothenburg and analyzed by means of LC-MSMS (001–04 Method for Blood Plasma Analysis).

### Evaluation of Potency and Efficacy of AZD5634 in CF Preclinical Systems

#### Primary CF HBEC culture studies.

Using previously described techniques (2, 37), epithelial cells were harvested from excised lung tissue derived from CF donors homozygous for F508del CFTR, expanded, and grown until confluency was attained. The cells were then seeded on 6.5-mm-diameter, 24-well Transwell permeable membrane supports coated with NIH 3T3 fibroblast unconditioned media (0.5 × 10^6^ cells/filter; Corning Inc., Corning, NY) and further allowed to mature in differentiating media (PneumaCult ALI, STEMCELL Technologies, Cambridge, MA) for a minimum of 4–5 wk until fully differentiated. To stimulate fresh mucus secretion and formation of an even ASL layer, the cells were washed 48–72 h before experimentation with PneumaCult ALI media (80 µL), added apically, and then aspirated following 30–45 min incubation at 37°C. The washing process was repeated with a 15-min incubation period and the mucus layer was allowed to reestablish, after which the cells were treated with 1 µL of AZD5634 (1 µM, apical), alone and in combination with lumacaftor and ivacaftor (luma/iva; 3 µM/10 µM, basolateral). PBS (1% DMSO, apical) served as a vehicle control. Luma/iva was added 18 h before baseline addition (0 h) of AZD5634 and PBS, based on our prior experience demonstrating peak effect of this CFTR modulator at 24–48 h in this cell-model system (37). μOCT images were obtained at 0 h (pretreatment), and at 6 and 24 h posttreatment. Only passage 1 and 2 monolayers were used for these studies.

#### I_sc_ analysis.

CFTR-dependent ion transport electrophysiology was measured in HBEC cultures by *I*_sc_ using modified P2300 Ussing chambers (Physiologic Instruments, San Diego, CA) as previously described (37, 38). Increasing concentrations of AZD5634, benzamil, or vehicle, followed by the addition of benzamil (10 μM), benzamil plus low chloride (to establish a chloride gradient), forskolin (20 μM), and CFTRinh-172 (10 μM) were added sequentially. All chambers were maintained at 37°C and agonist stimulation was initiated within 15 min of placement in the chambers.

#### μOCT image acquisition and analysis.

μOCT imaging and analysis were conducted in vitro and ex vivo as detailed in previous publications (2, 39–41). Briefly, the μOCT system uses light reflectance to produce video-rate (40 frames/s and 512 lines/frame), high-resolution images (1 micron) captured in real-time and without disruption of sample tissue, permitting simultaneous interrogation of key parameters of airway microanatomy without the addition of exogenous particles or dyes. Following imaging of HBEC cultures and of excised trachea and lungs, images were assessed to obtain quantitative measurements of mucociliary transport (MCT) rate, ASL depth, and ciliary beat frequency (CBF). MCT rates were calculated by analyzing the distance traveled by native particles in the mucus layer over time across multiple frames. Direct geometric measurement of layer thickness (Image J software) yielded ASL depths. CBF was determined by using custom code developed in Matlab (Mathworks, Natick, MA) to quantify the reflectance of beating cilia by Fourier analysis. Results for each cell monolayer reflected an average of measurements from images obtained from four consistently selected regions of interest: two captured 1 mm from the filter periphery, at the 9 and 12 o’clock positions, and two near the center of the filter where the μOCT laser transversed the mucus raft at an ASL height comparable with the cell layer height on the Transwell membrane. The results for each trachea and lung sample reflected an average of measurements from images obtained from a minimum of six controlled points along the tracheal length, using the cranial end as a reference.

#### Mucociliary evaluation in a rat CF model.

Six-month-old *Cftr*-knockout rats (female and male) were treated with seven 200-μL doses of either 54 µM AZD5634 or vehicle control, delivered by oropharyngeal aspiration, over 4 days (two doses daily for 3 days and a single dose on the 4th day, 2 h before euthanasia). Rats were euthanized by intraperitoneal injection of pentobarbital sodium (Fatal Plus, 100 mg/kg), and trachea and lungs were excised en bloc and dissected along the dorsal surface from the larynx to the terminal end of the main stem bronchi. Subsequently, the trachea and lungs were placed on DMEM media-soaked gauze and allowed to equilibrate to physiological conditions (37°C, 5% CO_2_, 100% humidity) for 30 min before μOCT imaging, described earlier.

### Statistical Analyses

#### In vitro healthy system assays.

All data are presented as means ± SE. Values of *n* refer to the number of cultures used in each group as appropriate. For normally distributed data, paired or unpaired Student’s *t* tests were used. For data that were not normally distributed, Mann–Whitney *U* test and Wilcoxon-matched pairs test were used. For comparisons of multiple groups, ANOVA tests were used followed by Tukey–Kramer multiple comparisons test (parametric) or ANOVA followed by Kruskal–Wallis test (nonparametric). For experiments using primary HBECs, a minimum of four different donors supplied cultures for each experiment. For the Ussing chamber data, unconstrained concentration–response curves were fitted using an in-house Microsoft Excel add-in package by nonlinear, four parameter, logistic regression analysis. From these curves, pEC_50_ and *E*_max_ values were taken from which mean values were calculated.

#### In vivo healthy system models.

Rat electrolyte data were analyzed by means of repeated-measures ANCOVA (SAS v.9). For MCC evaluations in sheep, slopes representing the rate of clearance and areas under the curve (AUCs) were compared, although due to the low number of sheep per group (*n* = 2) adequate statistical analysis of the data was not possible (Student’s *t* test between individual slopes for treatment group vs. control indicated significant differences, but only descriptive statistical analysis has been described and an individual data shown, with the averages given as a solid line).

#### In vitro and in situ experiments in CF systems.

Descriptive statistics were calculated and means compared using unpaired or paired *t* tests, or ANOVA, with post hoc analyses conducted for multiple comparisons where appropriate. For analyses of functional anatomy of en bloc trachea, a mixed linear model was used that corrected for within-animal correlation. All tests were two-sided and were computed using GraphPad Prism (GraphPad Software, Inc., La Jolla, CA). *P*-values <0.05 were considered significant. Statistics are reported as means ± SE, unless indicated otherwise.

For all experiments including data analysis, randomization and blinding to treatment groups were applied to the extent possible, according to Good Laboratory Practice.

### Study Approvals

The electrolyte handling rat model was approved by the local Ethical committee in Gothenburg (149/2013, approved site number 31–5373/11). MCC studies in healthy sheep were approved by the Mount Sinai Medical Center Animal Research Committee (IACUC No. 20-03-A-03). Approval for the use of human CF cells was obtained from the University of Alabama Birmingham (UAB) Institutional Review Board (IRB No. X150323006) and animal studies using CF rats were approved by the UAB Institutional Animal Care and Use Committee (IACUC No. 20344). All approvals were obtained before the initiation of all studies.

## RESULTS

### AZD5634 Potently Inhibits ENaC and Efficaciously Increases ASL in Healthy ALI Cultures

As pharmacological properties regarding selectivity are crucial for the development of ENaC inhibitors, we first assessed the properties of AZD5634 through a standard battery of assessments. AZD5634 had a greater selectivity and secondary pharmacology profile with its main hits at the α1 A receptor (0.2 µM) and DAT (0.91 µM), whereas Nav1.5 (>33.3 µM), Nav1.2 (>33.3 µM), hERG (13% inhibition at 10 µM), M2 receptor (>100 µM), and β2 receptor (>100 µM) were essentially unaffected (Millipore & Cerep, binding assays of 124 and 154 targets, respectively). In a separate set of experiments of mucus tracking in the CF piglet trachea and mouse ileum and trachea (35), it was shown that AZD5634 also blocks sodium/hydrogen exchangers, specificity unknown.

**Figure 1.**
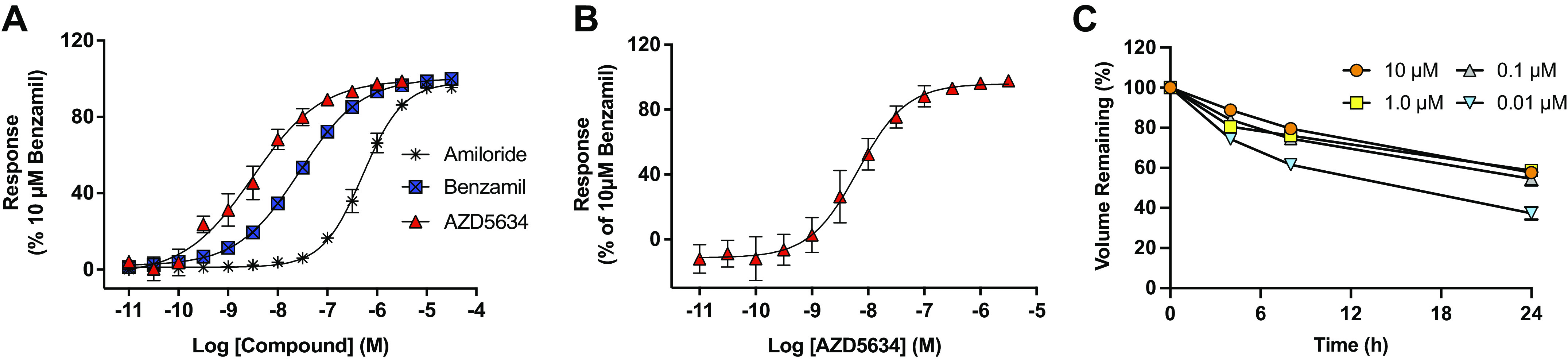
AZD5634 is a potent and durable inhibitor of ENaC in human, sheep, and rats. *A*: effective concentration (EC) curves for AZD5634 (EC_50_ 3.8 nM, *n* = 6 replicates), benzamil (EC_50_ 21.9 nM, *n* = 13 replicates), and amiloride (EC_50_ 0.454 µM, *n* = 2 replicates) in the Ussing chamber assay on normal human bronchial epithelial cells (HBECs) in air-liquid interface (ALI) cultures from healthy donors. All traces were normalized to 100% for the final dose of 10 µM benzamil following the cumulative concentration of the tested drugs. *B*: EC curves for AZD5634 (EC_50_ 5.5 nM, *n* = 5 replicates) in the Ussing chamber assay on ALI bronchial epithelial cells from normal sheep. *C*: duration of effect by AZD5634 on airway surface liquid (ASL) in ALIs from human bronchial epithelial (HBE) cells determined by gravimetric method indicates a duration of fluid-retaining effect >24 h by concentrations of 0.1 µM and above (*n* = 6 replicates). Means ± SE. ENaC, epithelial sodium channel.

We evaluated the potency and efficacy of AZD5634 in primary bronchial epithelial ALI cultures from healthy (non-CF) human donors and healthy sheep using *I*_sc_ as the readout. In HBEC cultures, AZD5634 caused a rapid decrease in *I*_sc_ (IC_50_ = 3.8 nM, *n* = 6), with a potency shift of 2.5 log units versus amiloride (IC_50_ = 0.45 µM, *n* = 2), and 1 log unit versus benzamil (IC_50_ = 21.9 nM, *n* = 13; Fig. 1*A*). No additional effect was seen when benzamil was added, suggesting the specificity of AZD5634 in targeting ENaC as a cause for the *I*_sc_ reduction observed. Consistent with HBEC results, the potency (IC_50_) of AZD5634 on inhibition of ENaC in bronchial epithelial cells derived from healthy sheep was determined to be 5.5 nM (*n* = 5, Fig. 1*B*). Using the gravimetric ALI assay, with its limitations, the duration of effect by AZD5634 in HBE ALI cultures was determined to be >24 h at concentrations of 0.1 µM and above (*n* = 6, Fig. 1*C*), which aligns well with the sheep and CF ALI data. To roughly estimate potency in normal rats, we used the effective concentration at inhibiting 50% of the potassium excretion (EC_50_) as a surrogate for ENaC blockade. The AZD5634 potency for rat ENaC was estimated to be 8.6 nM (*n* = 6; Supplemental Fig. S1). Together, these studies demonstrated similar potency of AZD5634 across species, as expected for this highly conserved ion channel.

### AZD5634 Is Efficacious and Durable on MCC in Normal Sheep In Vivo

**Figure 2.**
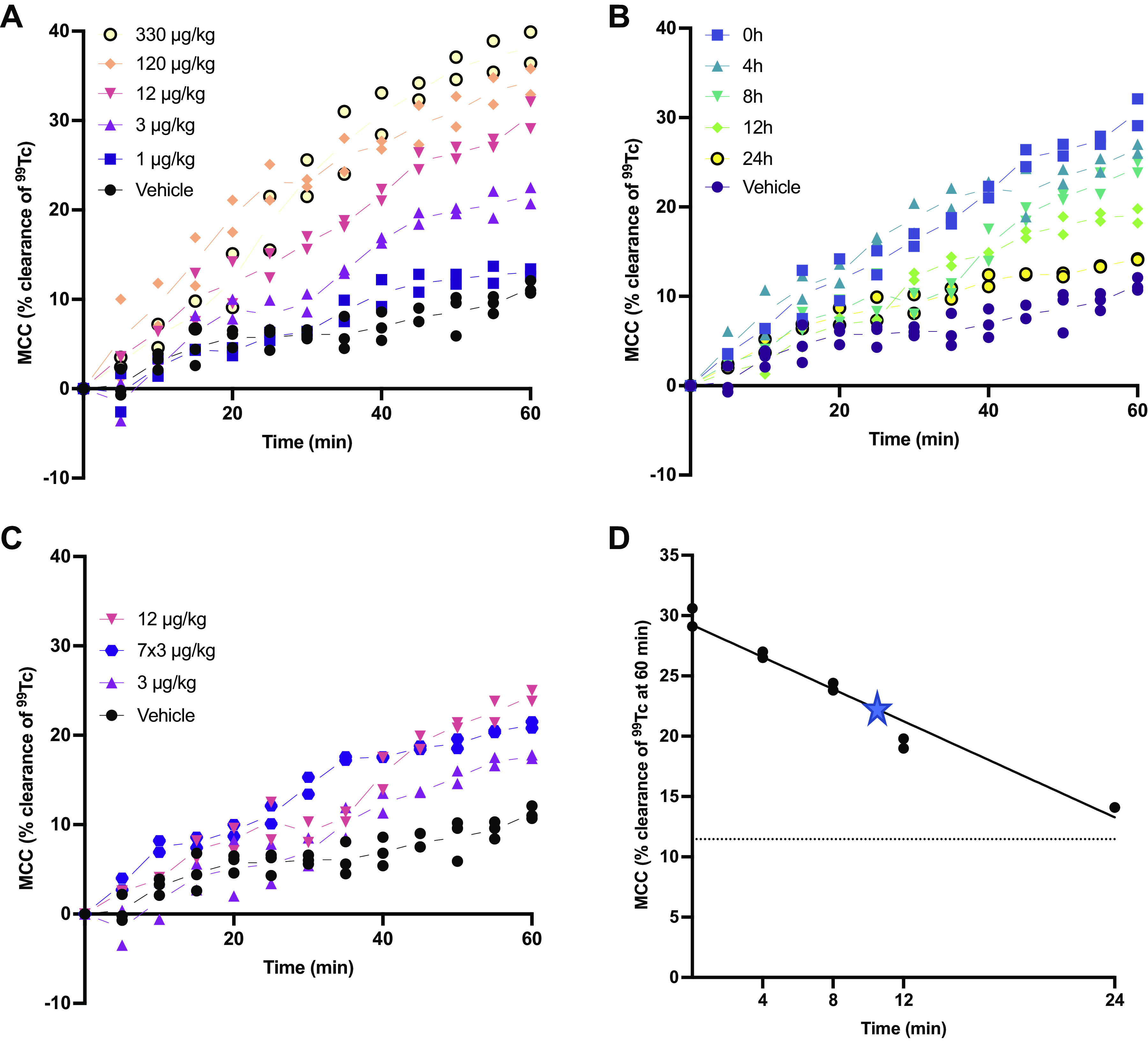
AZD5634 shows a dose-dependent increase of sheep MCC and a good duration of effect. *A*: for the dose-response evaluation, AZD5634 was administered just before the inhalation of ^99m^Tc. AZD5634 at 1–330 µg/kg demonstrates a dose-dependent increase of MCC (no stats performed due to the low *n*-numbers in the present model) with a max effect achieved at 120 µg/kg. *B*: for the evaluation of duration, the submaximal dose of 12 µg/kg AZD5634 was administered 0, 4, 8, 12, and 24 h before the ^99m^Tc administration. The retention of colloids was in all cases evaluated for 60 min after the administration of ^99m^Tc. *C*: repeated dosing of 3 μg/kg AZD5634 bid for 4 days (i.e., 7 doses) resulted in similar efficacy on MCC in sheep as 12 μg/kg 8 h after dosing. *D*: ^99m^Tc clearance of AZD5634 as a function of time as assessed by linear regression. The pharmacokinetic half-life of efficacy was estimated to be 9–10 h (marked with a star). Individual data are shown on the regression line, with the dotted line representing the mean clearance of vehicle-treated animals. *n* = 2–3 animals per group. MCC, mucociliary clearance.

To begin to determine the impact of AZD5634 on mucociliary function, and having established AZD5634 blocked ENaC in bronchial epithelial cells of sheep, we evaluated MCC in healthy female sheep, a model widely used to evaluate ENaC inhibitors. As shown in Fig. 2*A*, AZD5634 demonstrated a dose-dependent effect on MCC with max efficacy obtained at 120 μmoL/kg, as 330 μmoL/kg demonstrated a similar AUC. Then, 1 μg/kg and 3 μg/kg demonstrated some effect on MCC and 12 μg/kg demonstrated a submaximal effect, thus the latter dose was selected for further evaluation of duration of effect of the compound in this model. Most of the effect seen at 12 μg/kg of AZD5634 was retained for 8 h, with approximately half left at 12 h and another half of the remaining effect gone by 24 h, leaving some efficacy the day after dosing (Fig. 2*B*). Repeated administration daily for 4 days at 3 μg/kg bid showed that the effect magnitude was maintained compared with single-dose studies (Fig. 2*C*). The degree of MCC at 60 min versus time was used to calculate half-life of effect and was estimated at ∼10 h (Fig. 2*D*). Overall, these data indicated that AZD5634 augmented MCC in normal sheep in a concentration-dependent fashion, and was amendable to repeat dosing.

### AZD5634 Inhibits ENaC-Dependent *I*_sc_ in CF HBE ALI Cultures

**Figure 3.**
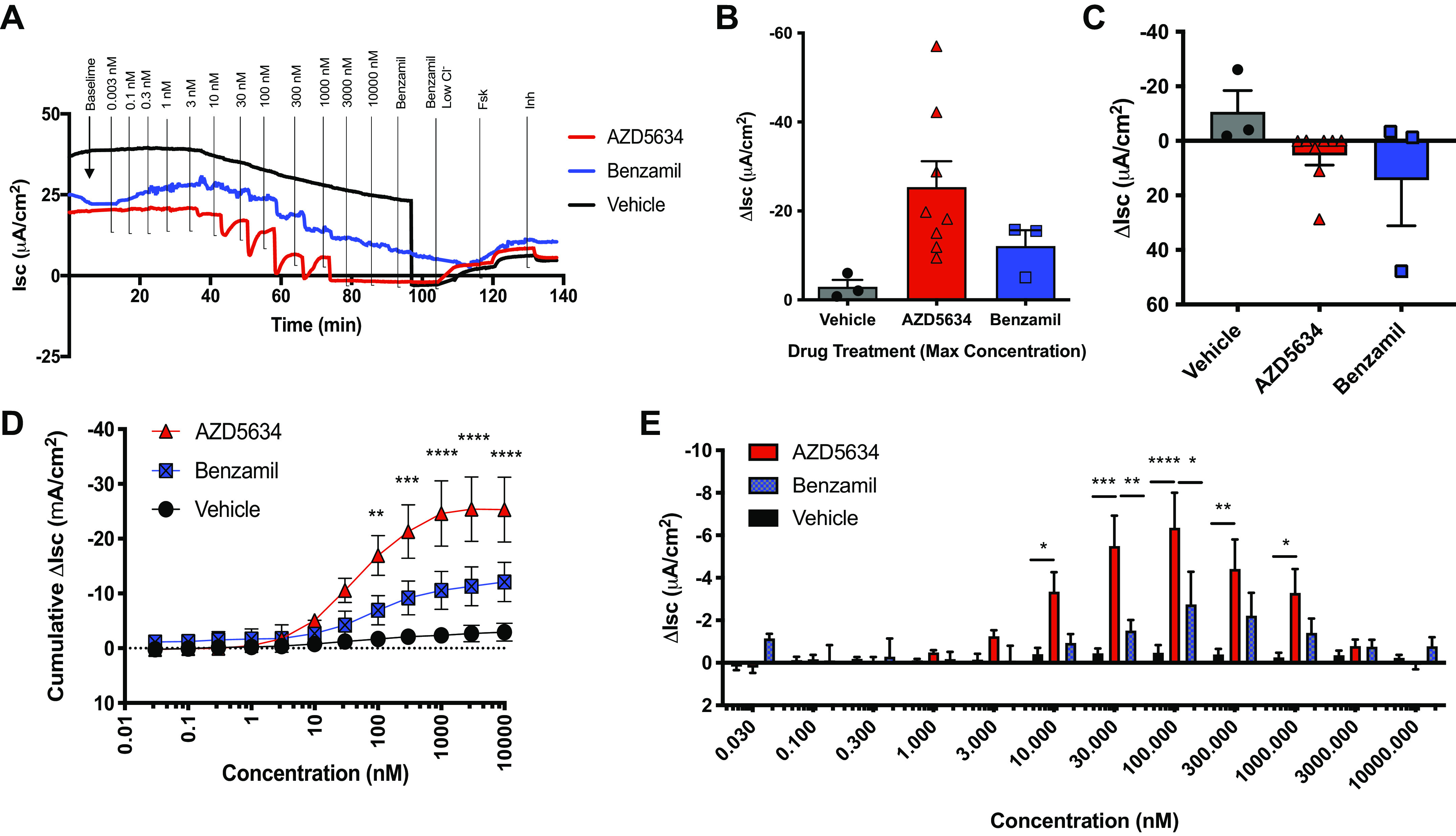
AZD5634 inhibits *I*_sc_ in F508del-homozygous HBEC cultures. *A*: representative tracing using modified Ussing chambers with sequential addition of increasing concentrations of AZD5634, benzamil, or vehicle, followed by the addition of benzamil, the establishment of a chloride gradient, and then the addition of forskolin and CFTRinh-172. *B*: quantitation of cumulative drug effect on ion transport with summary data showing change in cumulative *I*_sc_ (*C*) through maximum drug treatment concentration (10 µM). *D*: comparisons of change in *I*_sc_ elicited by AZD5634, benzamil, or vehicle at each concentration tested. Summary data showing change in *I*_sc_ following acute addition of benzamil (*E*). *n* = 8 monolayers for AZD5634, *n =* 3 monolayers each for benzamil and vehicle. Means ± SE, *P < 0.05, **P < 0.01, ***P < 0.001, and ****P < 0.0001. HBEC, human bronchial epithelial cell.

Based on the favorable potency and efficacy of AZD5634 in healthy systems, we proceeded with evaluations in HBE ALI cultures from F508del-homozygous individuals with CF. We first directly compared AZD5634 with benzamil in regard to efficacy and potency. A representative tracing depicting sequential addition of increasing concentrations of AZD5634, benzamil, or vehicle is shown in Fig. 3*A*. Quantitative analysis of cumulative drug effect (Fig. 3*B*) yielded a half maximal effective concentration (EC_50_) of 45.2 nM for AZD5634, 87.3 nM for benzamil, and 42.9 nM for vehicle. At the maximum concentration (10,000 nM), cumulative Δ*I*_sc_ with AZD5634 (−25.3 ± 5.9) exceeded vehicle (−2.9 ± 1.6, *P* < 0.05) and improved on benzamil (−12.1 ± 3.5, *P* = NS, Fig. 3*C*). In line with these findings, comparison of the potency of ENaC blockade between AZD5634, benzamil, or vehicle at each concentration tested revealed significant reductions in incremental *I*_sc_ with AZD5634 compared with vehicle at a dose range of 10–1,000 nM (Fig. 3*D*), peaking at 100 nM (−6.4 ± 1.6, *n* = 8, AZD5634 vs. −0.5 ± 0.4, *n* = 3, vehicle, *P* < 0.0001) where the effect also significantly surpassed benzamil (−2.7 ± 1.5, *n* = 3, *P* < 0.05). We further tested whether benzamil, added sequentially after AZD5634, could inhibit ENaC to levels beyond those seen with maximal concentration of AZD5634; no further appreciable reductions in *I*_sc_ were observed with AZD5634 upon acute addition of benzamil (Fig. 3, *A* and *E*), indicating completeness of ENaC blockade. Together, these studies indicated that AZD5634 effectively inhibited ENaC-mediated ion transport in CF primary cell cultures.

### AZD5634 Improves Airway Hydration and Mucus Transport in CF HBE ALI Cultures

**Figure 4.**
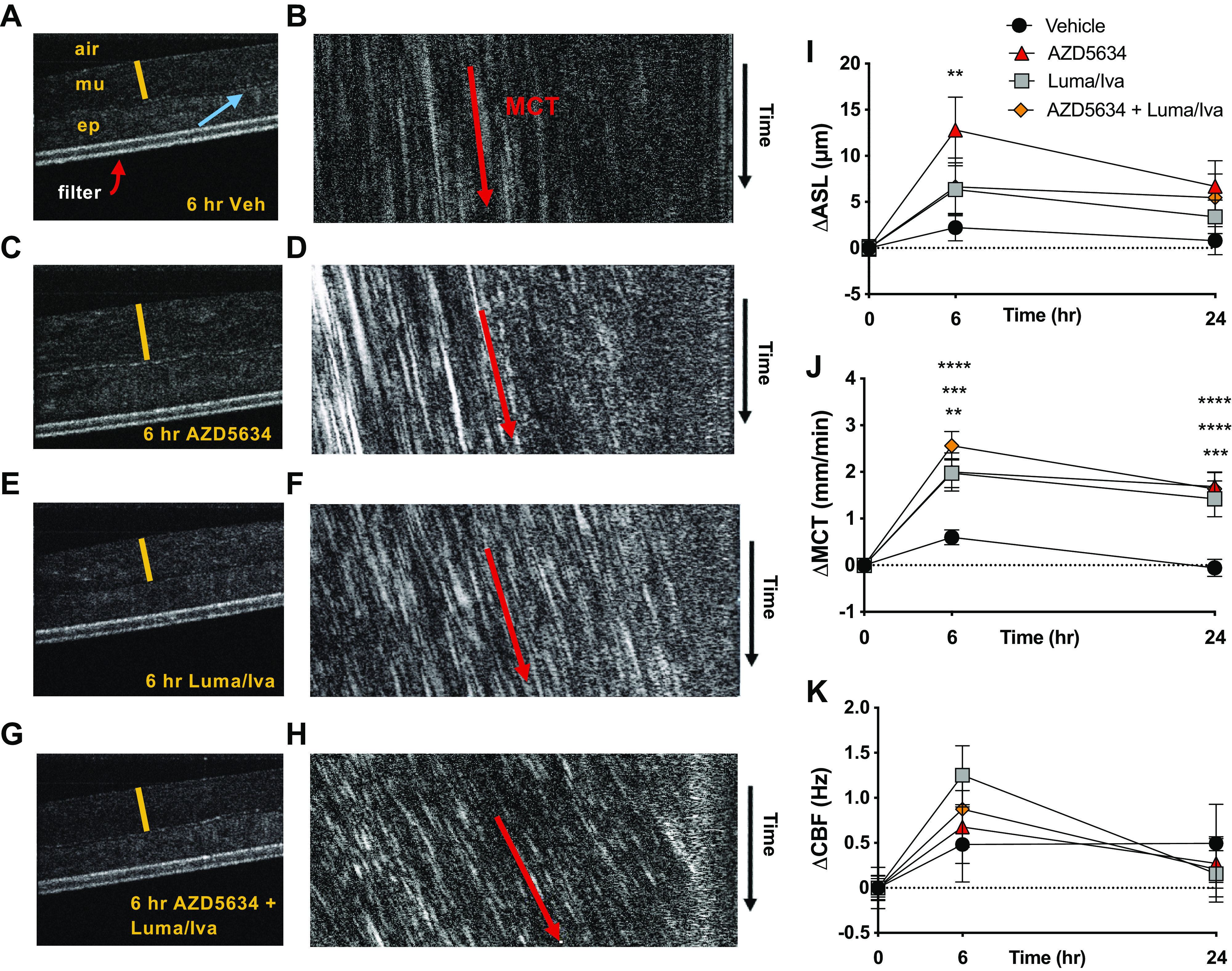
AZD5634 improves airway hydration and mucus transport in F508del-homozygous HBEC cultures. *A–K*: representative µOCT images depicting the effect of vehicle control (*A*), AZD5634 (*C*), lumacaftor/ivacaftor (luma/iva; *E*), and AZD5634 in combination with luma/iva (*G*) on airway surface liquid (ASL) depth at 6 h. (m) mucus, (ep) epithelium, blue arrow = cilia, red bar = ASL depth. *B and D–F*: images were resliced for calculation of mucociliary transport (MCT) rate, which was measured by projecting a cross-sectional line through the mucus through time. The slope of the particle trajectories indicated velocity. Change from baseline in ASL depth (*I*), MCT rate (*J*), and CBF (*K*) was assessed at 6 and 24 h post treatment. *n* = 17–18 monolayers per condition from a total of three different CF donors. Means ± SE, **P < 0.01, ***P < 0.001, and ****P < 0.0001. CBF, ciliary beat frequency; HBEC, human bronchial epithelial cell.

Continuing our evaluation in F508del-homozygous HBECs, we next assessed the functional impact of reduced ENaC activity with AZD5634 in this model using μOCT imaging. This technique enables simultaneous, real-time analysis of the effect of pharmacological manipulations on airway hydration, mucus transport, ciliary beating, and other mucus parameters, as we have shown previously in CF models (39), and provides a complementary approach and validation to gravimetric studies, noting the inherent limitation of using disparate assays across models. For these studies, HBECs were pretreated with the CFTR corrector-potentiator therapy lumacaftor plus ivacaftor for 18 h before the addition of AZD5634 to ascertain whether ENaC inhibition had an effect on top of standard of care. Representative μOCT images from which data were derived are presented in Fig. 4, *A*–*H*. Relative changes, normalized to baseline levels, in ASL depth, MCT rate, and CBF over the treatment period are reported in Fig. 4, *I*–*K*, with absolute values described here. We observed significant improvement in the ASL depth (*P* < 0.01; Fig. 4*I*) at 6 h in AZD5634-treated cultures, with levels (24.8 ± 3.6 µm) that exceeded those seen with vehicle (15.2 ± 2.5 µm) or the CFTR corrector-potentiator therapy lumacaftor plus ivacaftor (Luma/Iva; 18.8 ± 2.6 µm) and that were not enhanced by cotreatment with Luma/Iva (20.5 ± 3.1 µm). MCT rates with AZD5634 were elevated (2.5 ± 0.5 mm/min, *P* < 0.05) in relation to those observed with vehicle (1.0 ± 0.2 mm/min), in line with studies that have demonstrated improved mucus transport with increased mucus hydration (42–44), but in contrast to others where this relationship is absent (2, 3, 45). Luma/Iva had a similar impact on MCT (2.4 ± 0.4 mm/min, *P* < 0.05 vs. vehicle) and enhanced the effect of AZD5634 (2.8 ± 0.4 mm/min, AZD5634 + Luma/Iva, *P* < 0.01). These significant effects were sustained throughout the 24-h treatment period (Fig. 4*J*). There was no meaningful effect of AZD5634 on CBF (8.6 ± 0.5 Hz) relative to vehicle (8.4 ± 0.4 Hz, NS), as also seen with normalized values (Fig. 2*K*). Separate experiments corroborated the efficacy of AZD5634 in improving ASL and MCT in primary CF HBECs and additionally demonstrated that AZD5634 compared favorably with benzamil (Supplemental Fig. S2). We note that Luma/Iva conferred greater improvements to MCT than to ASL, and changes in CBF followed this pattern, possibly because the beneficial effect of CFTR-dependent anion secretion may be particularly useful in restoring mucus transport in this system, as opposed to inhibiting fluid absorption. Overall, these findings in primary bronchial epithelial cells suggested the potential of AZD5634 as a monotherapy and as an adjuvant therapy to CFTR correctors and potentiators for treatment of the mucus clearance defect in CF.

### AZD5634 Did Not Significantly Alter Airway Hydration or Mucus Transport in *Cftr*^−/−^ Rat Trachea In Situ

**Figure 5.**
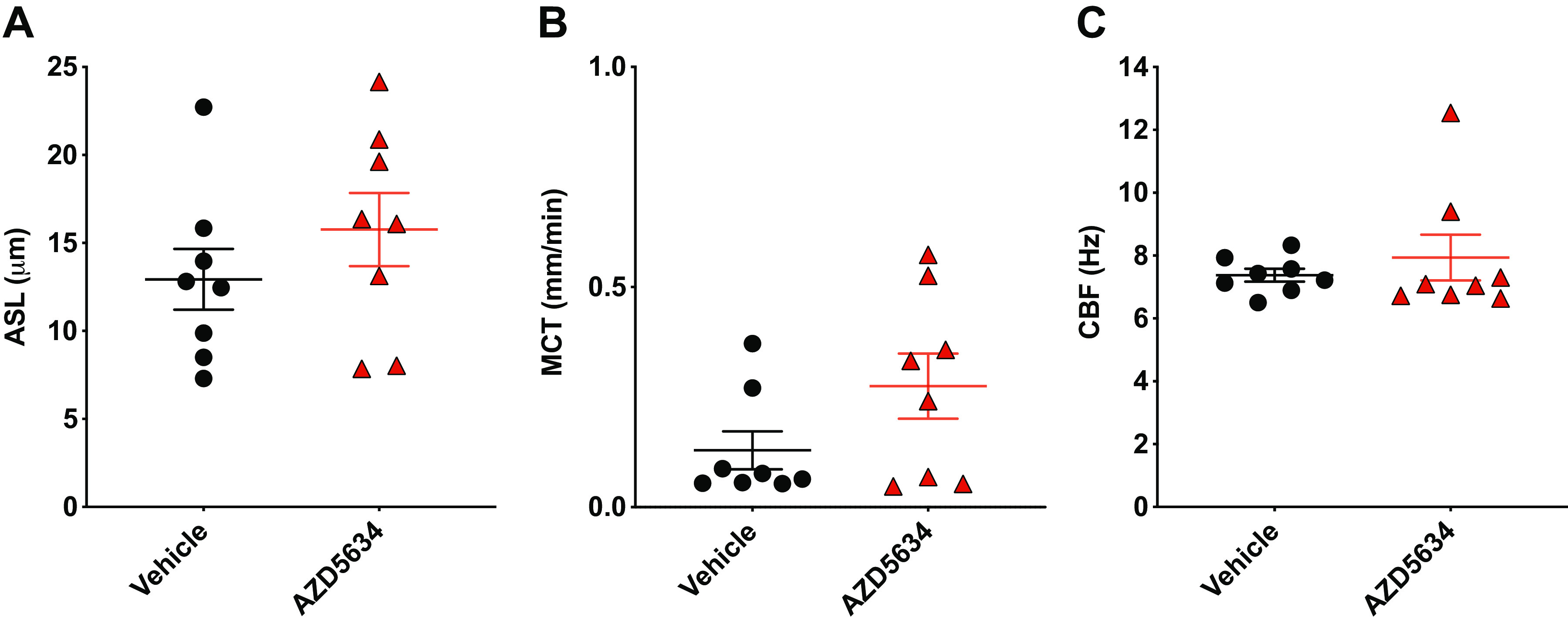
AZD5634 does not significantly alter functional anatomy in *Cftr*^−-/−^ rat trachea and lungs in situ. Airway surface liquid (ASL) depth (*A*), mucociliary transport (MCT) rate (*B*), and ciliary beat frequency (CBF; *C*) as determined by µOCT analysis of trachea and lungs excised en bloc from *Cftr*^−-/−^ rats treated with AZD5634 or vehicle control. *n =* 8 animals per group. Means ± SE.

We extended our μOCT analysis in situ in *Cftr*^−/−^ rats, a model that develops multiple features of the CF airway mucus defect including delayed mucus transport and a dehydrated airway (31). Animals were treated with AZD5634 or vehicle via oropharyngeal aspiration before euthanasia and en bloc excision of trachea, main-stem bronchi, and lung parenchyma together, a technique we have used previously that allows for inclusion of the role of peripheral fluid transport to the central airways (39). We found that ASL depth and MCT rate were moderately, but not significantly, elevated in AZD5634-treated animals (15.8 ± 2.1 µm, ASL, Fig. 5*A*; 0.3 ± 0.1 mm/min, MCT, Fig. 5*B*) compared with those treated with vehicle (12.9 ± 1.7 µm, *P* = 0.21 ASL; 0.1 ± 0.0 mm/min, *P* = 0.12 MCT). Ciliary beating also was not significantly altered (7.9 ± 0.7 Hz, AZD5634 vs. 7.4 ± 0.2 Hz, vehicle, *P* = 0.36, Fig. 5*C*). Additional experiments evaluating in vivo treatment effect on trachea that were excised in isolation yielded similar results, as did assessments upon pharmacological addition directly to excised trachea (data not shown). As AZD5634 has recently been demonstrated to promote mucus bundle detachment through isoform-specific sodium/hydrogen exchange (NHE) inhibition independent of ENaC (35), it is possible that insufficient NHE inhibition due to the absence or low expression of key NHE isoforms in the airways of *Cftr*^−/−^ rats may have contributed to this lack of translation in vivo; however, this is yet to be studied and is not illuminated by the μOCT technique, which is focused on the nonstrand mucus-ASL layer and does not allow for quantification of mucus bundle detachment that would be informative on NHE dependence. In sum, these findings suggested that treatment with AZD5634 was not sufficient to overcome the mucus clearance impairment in *Cftr*^−/−^ rats.

## DISCUSSION

Here, we present the preclinical evaluation of AZD5634, a small-molecule inhibitor of ENaC function intended to restore ASL depth and augment MCC in people with CF. In both non-CF and CF airway cells, AZD5634 exhibited potent and sustained inhibition of amiloride-sensitive current (*I*_sc_) that was 2 to 3 logs more potent than amiloride. Consequently, AZD5634 diminished ASL fluid resorption in non-CF HBE cells. ENaC inhibition by AZD5634 was nearly complete in sheep bronchial epithelial cells, and in normal sheep, this demonstrated dose-dependent improvement of mucus clearance in vivo, with a peak effect of 30% augmentation and a pharmacodynamic (PD) half-life of ∼10 h. In primary HBE cells derived from CF donors, AZD5634 also exhibited micromolar potency that exceeded the effect of the control ENaC inhibitor benzamil. This translated to improved ASL depth and accelerated MCT rates that compared similarly to the effects of the CFTR modulator combination lumacaftor plus ivacaftor in CF cells derived from F508del homozygous individuals. Despite these prominent effects in vitro, neither ASL depth nor MCT rates were increased in CF rats nebulized AZD5634. These studies demonstrate that while AZD5634 provides target engagement of ENaC in CF epithelial cells and consequently provides a downstream effect on ASL depth and MCT in vitro, this was not sufficient to definitively increase MCT in a CF animal model that exhibits delayed mucus clearance (31).

We do not know the reason why improved MCT was not demonstrated in CF rats after in vivo treatment with AZD5634, noting some favorable trends were present. One clear possibility is that the dose, delivery, potency, or efficacy were insufficient to augment mucus clearance, even though some ENaC inhibition was observed. Additional studies evaluating a range of doses or durations may rule out this possibility, although we have noted full target engagement at 1 μM with a duration of ∼ 24 h (human ALI cultures in vitro and sheep in vivo) and similar potency across species (human, sheep, and rats), which suggests that 50 μM instilled should be sufficient to induce bioactivity. Our own sample size calculations suggested further studies were unlikely to result in definitive results with present resources. Noting CF rats do not exhibit elevated amiloride-sensitive currents (46), even though they do exhibit delayed MCC (31), suggests that ferrets or pigs could be a more responsive model to ENaC inhibition and that CF rats are possibly not the best preclinical disease model in the evaluation of ENaC inhibitors if the primary objective is to ascertain bioactivity of the ENaC inhibitor, rather than impact of ENaC inhibition of CF pathophysiology in the context of a model without hyperactive ENaC. It is plausible that augmented airway hydration through ENaC inhibition was not sufficient to induce mucus detachment, an event that requires bicarbonate and chloride secretion to detect in tissue samples (2, 3, 31), and this may have hampered improvements in CF MCC in vivo. Recent studies conducted in parallel with the current experimentation showed AZD5634 also blocks sodium/hydrogen exchangers (35) and could augment mucus detachment in mice intestine and swine trachea by alkalinizing the airway surface; whether this also occurred in CF rats is unknown, but does not appear to have been sufficient to augment MCT as detected by μOCT. We elected to use the in situ technique for μOCT studies, with the lung and trachea en bloc, rather than analysis following ex vivo excision, as this is anticipated to capture the contribution of the small airways as mucus and fluid are propelled to the trachea from the distal lung, where mucus stasis is thought to be initiated in CF, noting this technique does not quantify transport rates of adherent mucus strands. This method was used successfully for a mucolytic agent that also was effective in in vitro studies (39). Nevertheless, the sensitivity and specificity of the technique across a range of therapies and the translation to human CF pathophysiology has yet to be established.

Subsequent studies of AZD5634 in the clinic showed that AZD5634 acutely inhibited amiloride-sensitive potential difference when perfused to the nasal surface (47), establishing target engagement in people with CF, as also demonstrated in CF HBE cells presented here. Unfortunately, upon single-dose inhalation conducted within the same study, AZD5634 did not demonstrate augmented MCC measured by clearance of ^99m^Tc-labeled sulfur colloid protocols [NCT02950805, (47)], akin to findings in CF rats. It may also be that the effect of a single dose conducted within a small study was insufficient to impact MCC, also noting that MCC may lack sensitivity for small degrees of benefit, such as the effect of tezacaftor-ivacaftor in patients homozygous for F508del as opposed to the effect of ivacaftor in individuals with the G551D CFTR mutation (48, 49). As the evaluation omitted multidose administration that may be necessary to achieve adequate drug delivery to the mucus-impacted CF lung, this may also have contributed to the negative clinical evaluation. Nevertheless, the lack of MCC effect dampened enthusiasm for further development of AZD5634. Together the findings that ENaC could be inhibited in vitro and in vivo, but did not substantially augment MCC in a CF animal model or people with CF suggest the possibility that ENaC inhibition may not be sufficient to restore MCC in certain CF conditions where mucus express is prominent or accumulated, noting constraints in definitive interpretation due to the small study.

Several small-molecule ENaC inhibitors and peptide blockers have failed successful development in people with CF (9, 22–25), although generalized conclusions cannot yet be ascertained as to the cause of these failures. In several cases, preclinical studies demonstrated inhibition of amiloride-sensitive current and improved ASL depth in non-CF and CF donors (26–29). Frequently, augmentation of MCC in normal sheep has also been shown, as is the case for GS-5737 (30), camostat (26), Parion-552-02 (27), P-1037 (29), and other ENaC small-molecule inhibitors that failed in clinical development. Potential reasons include the inability to achieve concentrations and target engagement necessary to exhibit efficacy while also avoiding systemic absorption, challenges with drug delivery to the epithelium where adhesive CF mucus may block delivery to the small airways, and reduced pharmacological activity of the agents once inhaled due to instability or metabolism. Taken together, these studies point to important considerations that should be evaluated in the future. As we have previously advocated, future development efforts need to include measurement of ENaC activity and MCC in patients to assist with data interpretation and assure target engagement at the concentrations evaluated (9).

Although the data presented here substantiate conclusions, important limitations should be recognized. The studies in sheep showed clear bioactivity, but were not evaluated in animals that have inhibited CFTR function or neutrophil elastase-induced epithelial dysfunction, and thus may be misleading as to what could occur in a CF model. Although CF rats exhibit delayed MCT, and have the propensity for responding to pharmacological agents that improve MCT, as opposed to the porcine and ferret models, they do not exhibit spontaneous lung disease (50, 51), nor hyperactivated ENaC activity (46). Thus, the nature of the MCT defects in rats may differ from adult patients with CF where chronic infection is prominent. Although we interpret the degree of change in MCT as negative, the positive trend may indicate the potential for efficacy; power calculations made such additional studies beyond the scope of the current effort. In addition, as ENaC is also expressed in lung capillaries, it is possible that its inhibition could have less desirable effects in response to lung injury and consequent pulmonary edema (52, 53).

In summary, we show that the ENaC inhibitor AZD5634 inhibited amiloride-sensitive currents, augmented ASL depth, and accelerated MCT in normal sheep and CF in vitro models, but this did not translate to improved MCC in CF rats, a finding also demonstrated in a single-dose inhalation study in people with CF. Future ENaC agents should take advantage of CF animal models that exhibit abnormal mucus clearance, as this may have a better chance of successful translation to the clinic and test whether augmented hydration alone via ENaC inhibition is sufficient to improve MCC in people with CF.

## DATA AVAILABILITY

Data will be made available upon reasonable request.

## SUPPLEMENTAL DATA

10.6084/m9.figshare.17021939.v1 Supplemental Figs. S1 and S2: https://doi.org/10.6084/m9.figshare.17021939.v1.

## DISCLOSURES

A.Å., M.H., J.R., B.L., C.W., and A.M. are employees at AstraZeneca Gothenburg, Sweden. None of the other authors has any conflicts of interest, financial or otherwise, to disclose.

## AUTHOR CONTRIBUTIONS

A.Å., E.F.L., and S.M.R. conceived and designed research; R-J.S., J.E.P.L., N.K., A.T.A., E.B., J.R., B.L., C.W., and J.S. performed experiments; A.Å., E.F.L, R-J.S., J.E.P.L., N.K., A.T.A., E.B., L.E., J.R., C.W., and S.M.R. analyzed data; A.Å., E.F.L., and S.M.R. interpreted results of experiments; A.Å., E.F.L., and J.R. prepared figures; A.Å. and E.F.L. drafted manuscript; A.Å, E.F.L., R-J.S., J.E.P.L., N.K., A.T.A., E.B., L.E., M.H. J.R., B.L., C.W., A.M., J.S., and S.M.R. edited and revised manuscript; A.Å., E.F.L., R-J.S., J.E.P.L., N.K., A.T.A., E.B., L.E., M.H., J.R., B.L., C.W., A.M., J.S., and S.M.R. approved final version of manuscript.
